# A case report of thoracolumbar paraspinal myopathy as the cause of camptocormia in a patient with atypical parkinsonism

**DOI:** 10.1186/s12883-017-0899-x

**Published:** 2017-06-23

**Authors:** Yoon Kim, Ahro Kim, Aryun Kim, Beomseok Jeon

**Affiliations:** Department of Neurology, MRC and Movement Disorder Center, Seoul National University Hospital, Parkinson Study Group, Seoul National University College of Medicine, 101 Daehak-Ro, Jongno-Gu, Seoul, 110-744 South Korea

**Keywords:** Camptocormia, Bent spine syndrome, Parkinsonism, Postural deformity, Dystonia, Paraspinal myopathy

## Abstract

**Background:**

Camptocormia is severe flexion of the thoracolumbar spine, exaggerated during standing and walking but minimized in supine position. Even though camptocormia is a relatively common condition during the course of Parkinson’s disease, there is ongoing controversy concerning its mechanisms. The most widely accepted and yet still disputed one is dystonia. However, based on myopathic changes observed in the paraspinal muscle biopsies of some PD patients with camptocormia, the attempt to attribute camptocormia to myopathy has continued. This case presents evidence for paraspinal myopathy as the cause of camptocormia in a patient with atypical parkinsonism.

**Case presentation:**

A patient presented with a relatively acute onset of camptocormia and new-onset back pain. Upon examination, she had asymmetric parkinsonism. Magnetic resonance imaging of the lumbar spine revealed alterations in muscle signal intensity in the right paraspinal muscles at the L1–2 level. In the presence of persistent back pain, repeat imaging done two months later showed diffuse enlargement and patchy enhancement of the paraspinal muscles on T1-weighted imaging from T4 through sacrum bilaterally. About fifteen months after the onset of camptocormia, she underwent ultrasound-guided gun biopsy of the paraspinal muscles for evaluation of focal atrophy of the back muscles on the right. The biopsy revealed unmistakable myopathic changes, marked endomysial and perimysial fibrosis of the muscles, and merely mild infiltration of inflammatory cells but no clues regarding the cause of myopathy. On account of persistent back pain and MRI results indicative of ongoing inflammation, she was prescribed glucocorticoid, which she refused to take. Now merely two and a half years after the onset of camptocormia, she is in Hoehn and Yahr stage 4.

**Conclusions:**

The coincidence of back pain with the appearance of camptocormia and the imaging and pathology findings supportive of myopathy give strong evidence for paraspinal myopathy as the cause of the deformity in this patient. When a patient presents with a relatively acute onset of camptocormia accompanied by back pain, the clinician should not overlook the possibility of myopathy of paraspinal muscles, which may be one of the few treatable causes of camptocormia.

## Background

Camptocormia, also called the bent spine syndrome (BSS), is severe, involuntary flexion of the thoracolumbar spine that is exaggerated during standing and walking but minimized in supine position. Recognized as a relatively common condition during the course of Parkinson’s disease (PD), it is occasionally observed in other parkinsonian syndromes such as multiple system atrophy. There is ongoing controversy concerning its mechanisms. The most widely accepted and yet still disputed one is dystonia. However, based on myopathic changes observed in the paraspinal muscle biopsies of some PD patients with camptocormia that cannot be explained by dystonia, the attempt to attribute camptocormia to myopathy has continued. This case presents evidence for paraspinal myopathy as the cause of camptocormia in a patient with atypical parkinsonism.

## Case presentation

**Fig. 1:**
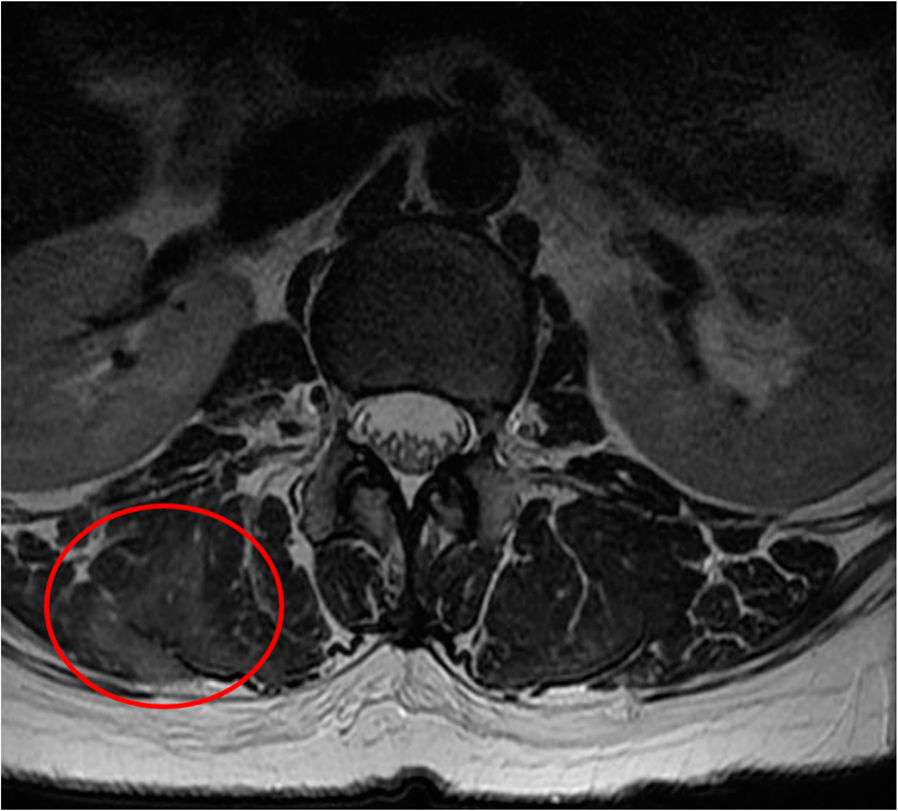
Non-contrast-enhanced L-spine MRI taken a month after the onset of back pain and camptocormia. Axial T2-weighted imaging shows alterations in muscle signal intensity in the right paraspinal muscles (*red circle*) at the L1–2 level

A 63-year old female was admitted to the neurology department with a chief complaint of progressive forward and leftward flexion of the spine that was exacerbated during walking but lessened on recumbent position. Two years earlier, she had been diagnosed with a mild degree of lumbar spinal stenosis at L3–4 and L4–5 levels by an orthopedic specialist. Even though she still experienced intermittent radiating pain from the left hip down to her left leg, she was still able to do mountain climbing and carry out her usual activities of living. One month prior to her admission to the neurology department, she started to notice progressive forward and leftward flexion of the back and new-onset lower back pain. She went back to the orthopedic specialist for medical advice. Non-contrast-enhanced magnetic resonance imaging (MRI) of the lumbar spine revealed alterations of muscle signal intensity in the right paraspinal muscles at the L1–2 level on T2-weighted imaging (T2WI) (Fig. 1), but no significant interval change of the known spinal canal stenosis. A facet joint injection of triamcinolone at right L4–5 level didn’t relieve her pain and it didn’t halt the progression of the spinal deformity. She was referred to the movement disorder clinic.

**Fig. 2:**
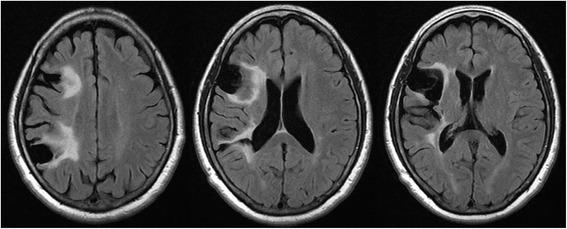
Brain MRI taken at the onset of camptocormia. Old infarcted lesions with tissue loss in the right pre-rolandic area, corona radiata, and postcentral gyrus are shown on FLAIR sequence

**Fig. 3:**
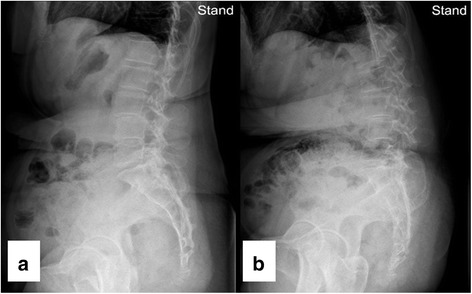
X-ray images of the lateral view of the spine on neutral standing position **a** one month before and **b** one month after administration of levodopa demonstrate progression of camptocormia despite medication

Upon examination, the patient did not have weakness, dysarthria or decreased sensation despite her history of infarction twenty years earlier in the territory of right middle cerebral artery (MCA) involving the right pre-rolandic area, corona radiata, and postcentral gyrus (Fig. 2). Muscle tone and deep tendon reflexes of the left extremities were unremarkable. Her sensation to proprioception was intact in all four extremities. She had action tremors of both hands, moderate bradykinesia and rigidity on her left side, and postural instability during turning. Her gait was not wide-based and she had no difficulty performing finger-to-nose and heel-to-shin. With definite evidence of parkinsonism, we gave her a trial of levodopa/carbidopa. Initially, levodopa equivalent daily dose (LEDD) of 150 mg per day was prescribed. Over a week, it was gradually increased to 600 mg per day. Even though the medication improved her gait by increasing step length without causing serious side effects, the forward flexion of the spine only became more apparent (Fig. 3). Serum creatine kinase (CK) was not checked at the time.

**Fig. 4:**
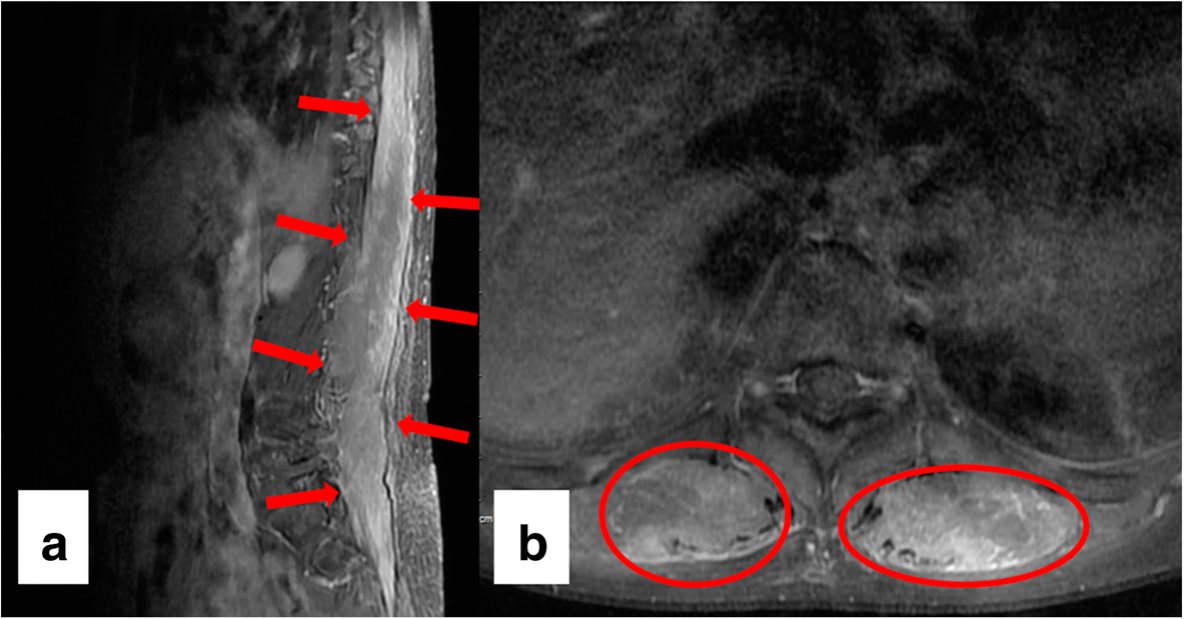
Spine MRI with gadolinium contrast taken three months after the onset of camptocormia. **a** Sagittal T1-weighted imaging reveals diffuse enlargement and patchy enhancement of the paraspinal muscles (*red arrows*) through the thoracic, lumbar, and sacral spine. **b** Axial T1-weighted imaging shows bilaterally increased signal intensity of paraspinal muscles (*red circles*) at the level of T10

She continued to experience severe back pain in the following months, besides which she developed erythematous pitting edema of and multiple bullae on both legs. She was subsequently admitted to the rheumatology. Her thyroid function was normal. CK was within normal limits. A repeat MRI scan of the spine, compared to the initial scan taken three months ago, showed diffuse enlargement and patchy enhancement of the paraspinal muscles on T1-weighted imaging (T1WI) from T4 through sacrum bilaterally (Fig. 4). Electromyography (EMG) demonstrated a mild to moderate amount of ongoing denervation potentials in the thoracic and lumbosacral paraspinal muscles but no evidence of myopathic motor unit action potentials in either the paraspinal muscles or the lower extremity muscles – the left tibialis anterior, peroneus longus, and gastrocnemius. Atorvastatin, which she had been taking since five years ago, was discontinued as the possibility of drug-induced myopathy could not be ruled out. At discharge, she was on opioids for unremitting lower back pain.

A year later she was re-admitted to the neurology department for evaluation of focal atrophy of the back muscles on the right. The pain had subsided considerably for a year now. MRI of the lumbar spine again confirmed the presence of non-specific myopathic changes of thoracolumbar paraspinal muscles. Patchy contrast enhancement along myotendinous or myofascial junction on T1WI and diffuse enlargement of thoracolumbar paraspinal muscles were still seen. But the extent of contrast enhancement and that of muscle enlargement were less compared to the MRI taken one year ago. She finally underwent an ultrasound-guided gun biopsy of the paraspinal muscles.

**Fig. 5:**
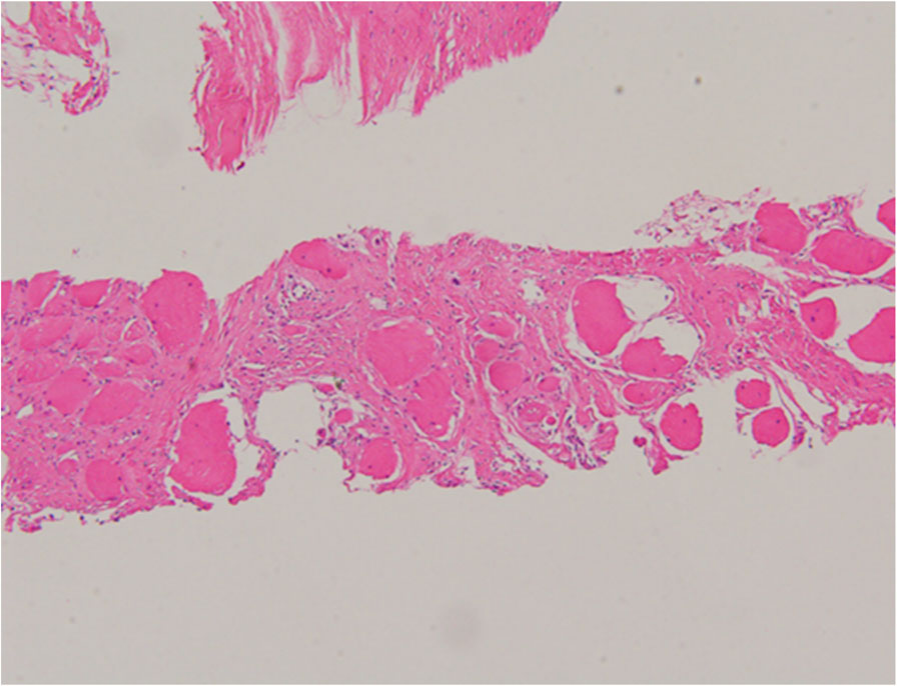
Paraspinal muscle biopsy. Hematoxylin and eosin staining of the specimen shows marked endomysial and perimysial fibrosis of the muscle fibers

The biopsy specimen contained myofibers with moderate size variation. Degenerated, atrophic and regenerating fibers were abundant and mostly round in shape. Atrophic myofibers were not angular in shape. The increase in the number of internalized nuclei was seen in many myofibers. There was marked endomysial and perimysial fibrosis of the biopsied muscles but merely mild infiltration of inflammatory cells in endomysial and perivascular space (Fig. 5). Overall, myopathic changes were evident, but the pathologist was unable to find clues with respect to the cause of fibrosis.

**Fig. 6:**
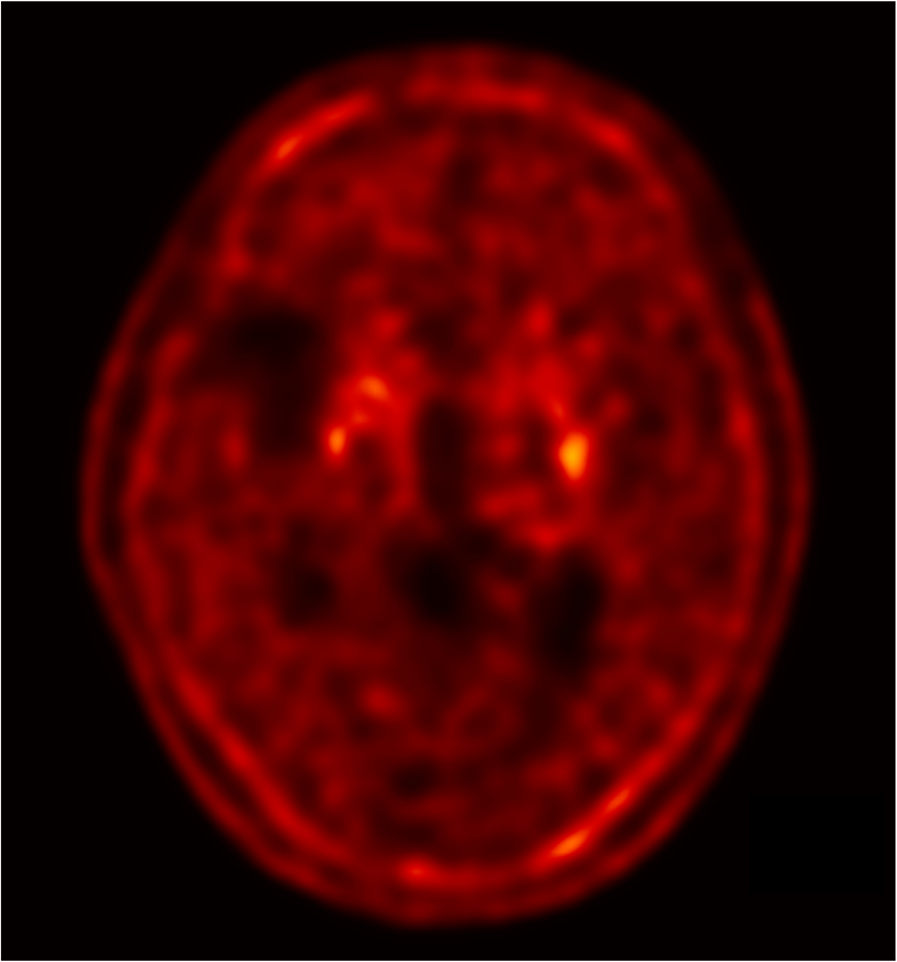
FP-CIT PET imaging. An axial image demonstrates severely decreased radiotracer uptake in both putamina. The caudate nuclei are not spared

Two years after the onset of camptocormia, as her clinical course was not consistent with idiopathic PD with camptocormia being the presenting symptom, fluorine-18 labeled N-3-fluoropropyl-2β-carboxymethoxy-3β-(4-iodophenyl)-nortropane (FP-CIT) positron emission tomography (PET) was done to confirm degenerative parkinsonism in this patient. The PET imaging revealed severely decreased radiotracer uptake in both putamina, even after taking into account the old infarct in the right MCA territory (Fig. 6). The bilateral caudate nuclei were not spared.

Since she continued to experience back pain, follow-up spine MRI was done a year after the biopsy. Findings suggestive of active inflammation – contrast enhancement on T1WI and hyperintensity of the paraspinal muscles on T2WI – were still present albeit to a lesser extent. Newly identified hypointense signals on T1WI and T2WI were indicative of fibrotic changes. On account of persistent pain and MRI results indicative of the presence of ongoing inflammation, she was prescribed glucocorticoid, which she refused to take. Now merely two and a half years after the onset of camptocormia, she is in Hoehn and Yahr stage 4. The patient is still on levodopa currently at the LEDD of 420 mg per day. She hasn’t developed supranuclear gaze palsy, freezing of gait, blepharospasm, or gait ataxia. She has urinary dysfunction – urinary frequency starting a year before the onset of camptocormic symptom along with documented evidence of urinary retention (post-voiding residual volume > 200 ml). However, aside from urinary dysfunction, she has not developed other symptoms or signs of autonomic dysfunction (e.g., orthostatic hypotension and constipation).

## Discussion and conclusions

Camptocormia is the stooped parkinsonian posture at its extreme. The condition appears to be more prevalent among those PD patients with a relatively long disease duration, high Hoehn and Yahr stage, [1] and akinetic-rigid form of parkinsonism more than the tremulous form [2]. The pathogenesis is still not understood fully.

The most conventionally cited, and yet still disputed hypothesis views camptocormia as a form of dystonia. The fact that camptocormia usually worsens while standing or walking is compatible with the definition of action-induced dystonia. In addition, in some patients, camptocormia is alleviated by maneuvers such as standing against a wall or wearing a backpack, which is comparable with sensory tricks seen in dystonia. On EMG, dystonia presents as co-contraction of agonists and antagonists. Biopsies of muscles affected by dystonia reveal no myopathological changes. However, the hypothesis of dystonia is still not firmly established as investigations into the neurophysiological basis for dystonia in camptocormia have not been thoroughly carried out yet.

Even more controversial is the attempt to attribute camptocormia to myopathy. With respect to the mechanism of myopathy, two concepts have been elaborated. The first concept considers camptocormia as primary BSS, in other words, a coincidental idiopathic myopathy entirely unrelated to PD itself [1]. On biopsy, primary BSS is characterized by unequivocal myopathic changes, a marked decrease in the number of myofibers, endomysial fibrosis, and fatty infiltration of paraspinal muscles [2]. Serum CK is elevated only in some cases and EMG reveals mostly myopathic changes, but neuropathic or normal findings in certain instances. MRI reveals selective and marked fatty replacement of the paravertebral muscles.

Within this framework of primary myopathy lies the concept of focal myositis of paravertebral muscles. A focal myositis is a localized, benign, and often self-limited inflammation of skeletal muscles usually of unknown origin that presents as a focal mass that undergoes rapid enlargement over a few weeks [3]. Its histopathology is marked by unmistakable myopathic changes and inflammatory cell infiltrates usually composed of lymphocytes. There have been only few case reports of BSS in PD so far that could be regarded as examples of true focal myositis, [4, 5] not myopathy of other or unknown etiologies.

The second concept within myopathy construes camptocormia as the primary problem of central proprioceptive dysregulation manifesting as secondary, nonspecific myopathic changes. The histopathological changes witnessed in paraspinal muscles from PD patients with camptocormia are remarkably similar to the findings of soleus muscle in rats after experimental Achilles tenotomy, [6] which interrupts the flow of information on muscle tension from the Golgi tendon organ through the polysynaptic reflex arc. Tenotomy leads to the following changes in muscles: core-like lesions in type 1 fibers characterized by sarcomere disorganization, reduced activity of oxidative enzymes such as succinate dehydrogenase, and increased activity of acid phosphatases [7]. These post-tenotomy changes are specific to tenotomy in otherwise intact muscles; these changes cannot be observed in muscles that had already been denervated. It appears irrelevant for the development of characteristic myopathic changes, whether the proprioceptive information flow is interrupted at the level of the Golgi tendon organ, the dorsal root ganglia, or at the supraspinal level [6]. The basal ganglia, involved in the control of axial muscle tone via proprioception, [8] may serve as a supraspinal control center of proprioception. Based on the concordance of myopathic changes, it may be reasonable to consider certain cases of camptocormia in PD as stemming from central proprioceptive dysregulation.

Then what is the cause of camptocormia in this patient with atypical parkinsonism? At least initially, dystonia could not be ruled out because the patient displayed nearly normal posture on initiation of gait that progressively became kyphotic as she continued to walk. However, now two and a half years after the onset of back pain, dystonia can be disregarded; the patient’s bent spine is not aggravated by standing or walking but is present to the same degree even in a sitting position. In contrast, there is unequivocal evidence in support of myopathy as the cause – coincidence of back pain with the appearance of camptocormia, MRI findings of active inflammation and chronic fibrotic changes, and myopathic changes on muscle biopsy despite the lack of myopathic EMG findings from paravertebral muscles. The biopsy results argue for primary idiopathic myopathy rather than a focal myositis or myopathic changes secondary to proprioceptive dysregulation.

Even though the hypothesis of myopathy of paraspinal muscles has been criticized for various reasons, this case suggests that at least in some patients with camptocormia, paraspinal myopathy plays a central role in its development. That merely fibrosis was noted in the biopsied muscles is not surprising given a period of more than a year had passed since the onset of back pain. Had the biopsy of paraspinal muscles been done at the onset of camptocormia, the nature of the myopathy could have been clarified. When a patient presents with a relatively acute onset of camptocormia accompanied by back pain, the clinician should not overlook the possibility of myopathy of paraspinal muscles, which may be one of the few treatable causes of camptocormia.

## Abbreviations

BSS: Bent spine syndrome
CK: Creatine kinase
EMG: Electromyography
FP-CIT: N-3-fluoropropyl-2β-carboxymethoxy-3β-(4-iodophenyl)-nortropane
MCA: Middle cerebral artery
MRI: Magnetic resonance imaging
PD: Parkinson’s disease
PET: Positron emission tomography
T1WI: T1-weighted imaging
T2WI: T2-weighted imaging
